# In Search of Pathogens: Transcriptome-Based Identification of Viral Sequences from the Pine Processionary Moth (*Thaumetopoea pityocampa*)

**DOI:** 10.3390/v7020456

**Published:** 2015-01-23

**Authors:** Agata K. Jakubowska, Remziye Nalcacioglu, Anabel Millán-Leiva, Alejandro Sanz-Carbonell, Hacer Muratoglu, Salvador Herrero, Zihni Demirbag

**Affiliations:** 1 Department of Genetics, Universitat de València, Dr Moliner 50, 46100 Burjassot, Spain; E-Mails: agatajak@gmail.com (A.K.J.); alexsanzc@gmail.com (A.S.-C.); 2 Department of Biology, Faculty of Sciences, Karadeniz Technical University, 61080 Trabzon, Turkey; E-Mail: zihni.demirbag@ktu.edu.tr; 3 Instituto de Hortofruticultura Subtropical y Mediterránea “La Mayora” (IHSM-UMA-CSIC), Consejo Superior de Investigaciones Científicas, Estación Experimental “La Mayora”, Algarrobo-Costa, 29750 Málaga, Spain; E-Mail: anabel@eelm.csic.es; 4 Department of Molecular Biology and Genetics, Faculty of Sciences, Karadeniz Technical University, 61080 Trabzon, Turkey; E-Mail: muratoglu@ktu.edu.tr

**Keywords:** *Thaumatopoea pityocampa*, pine processionary moth, PPM, transcriptome, iflavirus, cypovirus, rhabdovirus

## Abstract

*Thaumetopoea pityocampa* (pine processionary moth) is one of the most important pine pests in the forests of Mediterranean countries, Central Europe, the Middle East and North Africa. Apart from causing significant damage to pinewoods, *T. pityocampa* occurrence is also an issue for public and animal health, as it is responsible for dermatological reactions in humans and animals by contact with its irritating hairs. High throughput sequencing technologies have allowed the fast and cost-effective generation of genetic information of interest to understand different biological aspects of non-model organisms as well as the identification of potential pathogens. Using these technologies, we have obtained and characterized the transcriptome of *T. pityocampa* larvae collected in 12 different geographical locations in Turkey. cDNA libraries for Illumina sequencing were prepared from four larval tissues, head, gut, fat body and integument. By pooling the sequences from Illumina platform with those previously published using the Roche 454-FLX and Sanger methods we generated the largest reference transcriptome of *T. pityocampa*. In addition, this study has also allowed identification of possible viral pathogens with potential application in future biocontrol strategies.

## 1. Introduction

*Thaumetopoea pityocampa* (pine processionary moth) is one of the most important pine pests in the forests of Mediterranean countries, Central Europe, the Middle East and North Africa [1]. Pine processionary moth (PPM) larvae feed on the needles of pine trees and some other conifer tree species. PPM larvae build white winter nests that are easily discernible and thus provide unambiguous indication of species presence since no other organism produce similar structures in these tree species at that time of the year [2]. In large numbers they can severely defoliate trees, weakening them and making them more susceptible to attack by other pests or diseases, or to environmental stress caused by drought or excessive moisture [3]. PPM larvae not only cause significant damage to forest trees but are also responsible for dermatitis, ocular lesions and, more rarely, respiratory signs and anaphylactic reactions in humans and animals [1].

PPM is also one of the most important forest pests in Turkey. The larvae of this pest feed on coniferous species, *Pinus brutia*, *P. nigra*, *P. pinaster*, *P. pinea* and *Cedrus libani* in an area of over 1.5 million hectare [4]. Control of this pest is thus a necessity, and different approaches have been used, including mechanical, chemical and biological methods [5]. Management of the PPM is now largely based on the use of *Bacillus thuringiensis* subsp. *kurstaki* (Btk) preparations. Despite of its efficacy and environmentally friendly profile [6], Btk has rarely been used to control PPM in Turkey [7], while it is the most widely used method in control of PPM in the EU and in some Mediterranean countries [8,9]. In order to make the processionary moth control profitable, the economic analysis of using such control methods need to be tested. In a case study performed in Portugal, scientists examined the economic assessment of managing processionary moth in pine forests and concluded that pest management costs outweigh market revenues, making processionary moth control unprofitable for the forest owner in the short-term [10].This result clearly demonstrates that there is still need to find new control methods that do not require several inundative applications every season. Given the nature of the plant species to be controlled and the large areas to be treated, naturally occurring PPM pathogens that could be used in an innoculative strategy to keep the PPM populations under certain threshold would be of high interest. The occurrence of different pathogens including viruses [11], bacteria [11,12] and fungi [12] have been reported from PPM. Among them, viral pathogens are less likely to have an impact on non-target species in comparison to bacterial or fungal pathogens, as reported for the control of gypsy moth with *B. thuringiensis* treatments [13].

Massive parallel sequencing using Next Generation Sequencing (NGS) technologies is becoming a very useful approach for the discovery of novel microbes and viruses from animals and plants [14,15]. The technology allows for rapid, inexpensive, high throughput and accurate sequencing for identification of microbial and viral sequences derived from whole insects or specific tissues, and for viruses present at low titers that do not cause clear symptoms in the host [16]. To date, there are several studies on the discovery or whole genome sequencing of pathogens from insects or insect cell cultures by means of NGS [16,17,18,19,20,21,22,23]. It has to be mentioned, that the use of polyA-derived libraries biases the discovery of novel viral pathogens toward the RNA viruses. Using NGS technology, a transcriptome has been recently published regarding the comparison of two phenologically divergent populations’ of the PPM. In that study sequences were derived from insects reared in the laboratory for a while after field collections and sequence information did not report the discovery of new pathogens from PPM [24].

Here we have performed RNA sequencing using NGS in samples from PPM larvae collected from 12 different locations in Turkey with the aim of: (i) the establishment of a comprehensive larval transcriptome that could contribute to the study of different biological aspects of this pest; and (ii) the detection of possible viral pathogens naturally occurring in these insects that could provide novel tools for the biological control of that pest.

## 2. Materials and Methods

### 2.1. Sampling and RNA Isolation

**Figure 1:**
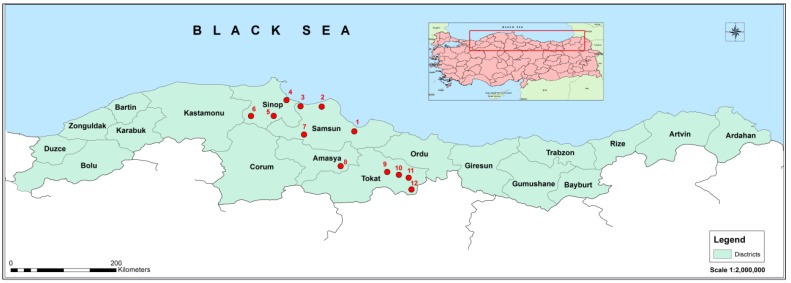
Map representing locations of insect sampling: (1) Samsun-Ankara highway; (2) Samsun-Alaçam; (3) Sinop vicinity; (4) Sinop-Gerze; (5) Sinop-Boyabat; (6) Sinop-Boyabat exit; (7) Samsun-Vezirköprü; (8) Amasya-Tokat highway; (9) Tokat-Sivas highway; (10) Tokat-Niksar highway; (11) Tokat-Reşadiye exit; and (12) Sivas-Koyulhisar.

The insect samples were collected in 12 different locations in Turkey (Figure 1). Larvae were sampled during the period of 2 January 2014 to 4 January 2014, in the L4 or L5 instar. Most of the larvae were collected during the procession stage, which occurs in the end of the larval stage in late winter and early spring. At least 15 larvae were collected from each geographical location (from five processions). The larvae were maintained in the laboratory for few days after collection and subsequently frozen at −80 °C, until being processed. Total RNA was isolated from various tissues of 8 individuals from each location. First, larvae were dissected and the following tissues and body parts were isolated under the binocular magnifying glass: (i) head (HE) capsules were cut with precision scissors; (ii) larvae were cut open along the body and guts (MG) were pulled up, separated from the rest and carefully cleaned from the content; (iii) fat body (FB) mass was separated from the other tissues; (iv) the integument (T) was cleaned from the leftover tissues by scraping them off. Immediately after dissection, tissues and body parts were placed in 1–2 mL Tripure Isolation Reagent (Roche) and homogenized mechanically. RNA was isolated from the homogenates according to the manufacturer’s protocol. Precipitation was carried out at −20 °C overnight and the precipitated RNA was dissolved in 100 µL of RNase-free water after being washed twice with 70% EtOH. RNA concentration was measured using Nanodrop spectrophotometer (Thermo Scientific). One microgram of RNA from each location was pooled for each tissue or body part, resulting in the four samples; gut (MG), fat body (FB), integument (T) and head (HE). These four RNA samples were additionally purified by using the RNeasy MinElute Clean up kit from Qiagen following the manufacturer’s protocol. RNA was eluted from the column in 100 µL of water and shipped to Macrogen Inc. (Korea) as EtOH/NaAc precipitate. All subsequent steps until sequencing were performed by Macrogen Inc. RNA integrity was verified on an Agilent 2100 Bioanalyser using the RNA 6000 Nano kit (Agilent Biotechnologies, Palo Alto, CA, USA). The RNA integrity numbers (RIN) were between 5.5 and 7.2. Low RIN values are most likely due to partial degradation of RNA during the freeze-thaw process prior to tissue extraction. Nevertheless, low RIN values might have also been obtained because insect 28S rRNA contains an endogenous break, and a total RNA profile differs considerably from a typical eukaryotic RNA. Since RIN algorithm includes also a calculation of 28S/18S rRNA ratio, the insect RNA RINs tend to be low, and it does not always imply RNA degradation [25].

### 2.2. Next Generation Sequencing and Raw Data Processing

Libraries were constructed and sequenced at Macrogen Inc. (Korea) using their standard procedure. For each RNA sample, poly-A based mRNA enrichment was performed and a cDNA library for 100 bp paired-end (PE) sequencing was generated using Illumina TruSeq™ RNA Sample Preparation kit v1 (Illumina, San Diego, CA, USA) following the manufacturer’s protocol. All four cDNA libraries were purified and concentrated, and their quality was estimated using Labchip GX from Caliper (Caliper Life Sciences, Runcorn, UK). The medium size of the cDNA library fragments was between 285–297 bp.

cDNA libraries derived from the four tissues and body parts of *T. pityocampa* were subjected to NGS with the Illumina HiSeq X Ten platform at Macrogen Inc. The Illumina paired-end reads (2 × 100 bp) were retrieved in their raw form from the sequencer and delivered by Macrogen as fastq files. The raw sequences were processed using the following protocols.

The whole sequence cleaning and assembly was performed with ngs_backbone pipeline developed at COMAV, Universitat Politècnica de València, Spain. First, low quality and short reads were removed, adaptors and primers were filtered, and 3' ends of reads with a quality less than 25 (Q < 25) were trimmed. Quality control was checked and compared to raw data by FastQC [26].

### 2.3. *De Novo* Transcriptome Assembly

Assembly was carried out using Trinity (version r20140413p1) software with default settings (kmer length 25) [27]. In order to obtain a more complete set of the genes expressed, we pooled the reads of the four libraries (MG, FB, T and HE), and we also included 454 and Sanger sequence reads from *T. pityocampa* collected in Portugal and kindly provided by H. Vogel from Max Planck Institute, Jena, Germany [24]. Four hundred fifty-four reads were previously processed (cleaned) as described above for Illumina reads. The raw Trinity assembly was further processed as follows. In order to reduce redundancy we used Cap3 program to combine highly similar transcripts, using overlap length cut-off of 200 and overlap percent identity cut-off of 99 (−o 200 -p 99) [28]. Subsequently, the isoforms were filtered by length (contigs shorter than 200 nt were discarded), complexity and expression levels. Trinity output gives large numbers of sequence isoforms, some of which are real splice variants, but many are chimeras. To identify the representative isoforms in Trinity, we mapped the reads to the assembled transcripts using RSEM [29] and removed transcripts expressed below the FPKM (fragments per kilobase per million fragments mapped) threshold of 1. After each step in the transcriptome assembly, the quality of the assembly was monitored by FastQC program. Each representative isoform will be used in subsequent functional annotation analyzes as a unigene.

### 2.4. Functional Annotations and GO Terms Assignment

Structural and functional annotation was performed by comparing the predicted genes sequences with public databases. Blastx (e-value cut-offs of 10^−20^) searches were performed against NCBI non-redundant vertebrate protein databases (release of 2014-05) as well as against UniProt/SwissProt, UniRef90, SilkDB, Flybase, and BeetleBase, prioritizing non-machine curated databases. Once a unigene had a solid blast hit in one of the databases, a description was built based on the description of the best hit. A bidirectional blast search comparison with SilkDB, Flybase and Beetlebase served to obtain a set of putative orthologs of *Bombyx mori*, *Drosophila melanogaster* and *Tribolium castaneum*, respectively, among the *T. pityocampa* unigenes.

Functional classification of the unigenes following the Gene Ontology scheme was performed using Blast2GO [30]. Whole transcriptome served as input to Blast2GO, to obtain the relevant GO terms for each sequence. Open reading frames (ORF) were predicted in the unigenes using ESTScan software [31].

### 2.5. Virus Sequences Detection, Identification and Phylogenetic Analyses

In order to detect possible viral pathogen sequences in the transcriptome of *T. pityocampa*, we used the following approaches. Blastx searches against the NCBI viral sequences were used to identify putative viral unigenes. Manual filtering was then applied in order to remove those sequences with similarity to functional domains (zinc finger domain, helicase domain, RNA binding domain, *etc.*) present in viral but also in not viral proteins and possibly not representing viral sequences. Moreover, unigenes representing the allelic variants or redundant unigenes from the same virus were also manually filtered based on additional Blastx searches.

Virus identification and family assignment was based on the sequence analysis and phylogenetic comparisons. Sequence and phylogenetic analyses were performed separately for the three putative viral related sequences. Sequence analyses were carried out by three different methods, depending on the sequences viral nature. Analyses corresponding to the genus *Cypovirus* were conducted by comparison of the nucleotide sequence of the RNA dependent RNA polymerase to confirm the affiliation of *T. pityocampa* virus to Cypovirus (not shown) and subsequently of the complete polyhedrin gene (Segment 10) [32,33,34] within the described members of the genus *Cypovirus*. Multiple sequences were aligned by MUSCLE alignment tool [35] in MEGA5 [36].

For the family *Iflaviridae*, analysis included the representative iflaviruses described so far, and *Drosophila C virus* (*Dicistroviridae*) as outgroup. Multiple sequence alignment of the predicted conserved domains of the RNA-dependent RNA polymerase amino acid sequence, were performed using PRALINE [37] and COBALT [38] software, for the best adjustment in the alignment.

Analyses corresponding to the family *Rhabdoviridae*, were carried out with some representative member from genus *Nucleorhabdovirus*, *Cythorabdovirus*, *Vesiculovirus*, *Ephemerovirus*, *Lyssavirus*, *Sigmavirus*, *Sprivivirus*, *Tupavirus*, *Tibrovirus* and *Perhabdovirus*, covering partial L protein sequences comprising 158 aa which included domain III [39] aligned by CLUSTAL W alignment tool [40] with the BLOSUM substitution model in MEGA5.

Alignments were examined and edited in Genedoc [41]. Best fitting models of molecular evolution were calculated in MEGA5. The selected substitution models were HKY, WAG+GI, and Blossum62+GI for Cypovirus, Rhabdovirus and Iflavirus putative members, respectively. Bayesian phylogenetic analyses were performed separately with BEAST v1.7.5 software [42], with a chain length of 10,000,000 sampling every 1000 trees. Outputs files were analyzed using TRACER v1.5 [43] and the final tree was summarized into the maximum clade credibility (MCC) phylogeny using TREEANNOTATOR v1.7.0 (beast.-bio.ed.ac.uk/TreeAnnotator), discarding the first 25% of sampled trees as burn-in. Final trees were visualized in FigTree v.1.4.0 [43]. All used sequences accession numbers are provided in the Table S1. Similar phylogenetic inference were conducted using the Maximum Likelihood method [44] for the family *Rhabdoviridae*, and Dayhoff [45] and JTT [46] models for the *Iflaviridae* resulting on similar trees topology, confirming the final trees’ structure.

### 2.6. RNAseq Expression Analysis and Tissue Specific Transcripts Identification

Cleaned reads corresponding to each of the four libraries (MG, FB, T, and HE) were mapped against the viral unigenes. For those redundant unigenes representing different variants of the same viral sequence, only the largest unigene was used for the mapping. Two different mapping methodologies were evaluated, the BWA-MEM [47] that follows the “*seed-and-extend*” aligner paradigm, and Subread [48], based on a novel strategy, called “*seed-and-vote*” obtaining slightly better mapping parameters (mapping quality and coverage) for the BWA-MEM method (data not shown). To check the relative abundance of each viral unigene in each of the analyzed samples, the output results were processed using SAMtools [49] and the coverage histograms were obtained from sequence alignment data files (BAM files) after normalizing by the millions of reads obtained in each library using BEDTools [50]. Coverage histograms were visualized with ggplot2 (*R* package) with *R* scripts personally customized [51].

## 3. Results and Discussion

### 3.1. T. pityocampa Transcriptome Assembly and Functional Annotation

The PPM is the biggest cause of defoliation in pine trees in Europe, but also in Central Asia and North Africa. Screening *T. pityocampa* for new viral pathogens forms part of the strategy to control this expanding pest. In order to increase the chances of detecting viral sequences we collected larvae of *T. pityocampa* from 12 distant geographical locations in the north of Turkey (Figure 1). After larval dissection, tissue pooling and RNA purification, samples were submitted for RNAseq using the Illumina platform (Pair-end sequencing). We obtained 111.5, 118.0, 114.7 and 118.4 million Illumina reads for gut, fat body, integument and head tissue samples, respectively. After sequence cleaning, we obtained 78.7, 87.7, 86.5 and 89.4 million reads for gut, fat body, tegument and head tissue samples, respectively. Phred quality score (Q score) was used to assess the accuracy of the reads. Q scores are defined as a property that is logarithmically related to the base calling error probabilities. For example, Q30 is equivalent to the probability of an incorrect base call 1 in 1000 times. Q30 is considered a benchmark for quality. In our sequencing project mean reads quality was 34 for all four libraries, and it improved to 36 after reads cleaning and processing (Table 1). All reads were deposited in the NCBI database (acc. Number: SRP050155).

**Table 1:** Sequencing features of the *T. pityocampa* transcriptome sequencing.

	MG	FB	T	HE
Nr of raw reads	111,505,976	117,988,640	114,664,330	118,378,516
Total sequence (Mb)	11,262,103	11,916,852	11,581,097	11,956,230
Sequence quality average	34	34	34	34
Nr of processed reads	78,680,476	87,668,878	86,497,974	89,424,098
Sequence quality average ^a^	36	36	36	36

^a^ After processing.

**Figure 2:**
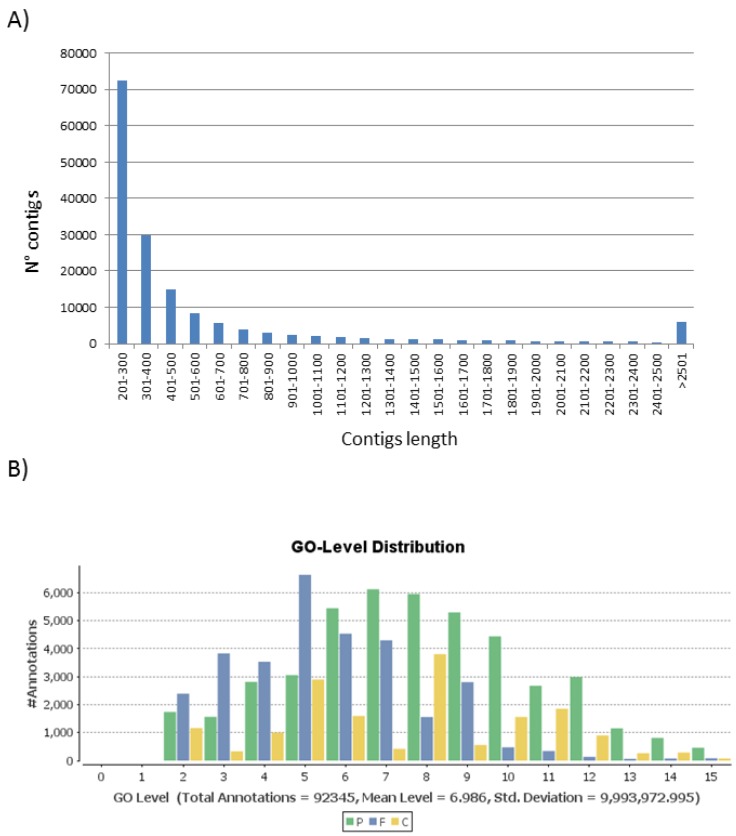
Transcriptome and functional analysis characteristics. (**A**) Length distribution of transcripts obtained in *T. pityocampa* transcriptome; (**B**) Gene ontology level distribution in *T. pityocampa* annotated unigenes in the three main GO categories: P- biological process, F- molecular function and C- cell component.

The transcriptome was assembled from Illumina reads including Roche 454-FLX and Sanger reads derived from a previous sequencing project from *T. pityocampa* from Portugal [24]. After assembly of the reads, 161,682 transcripts (152,669 unigenes after excluding alternative transcripts) were obtained (Table 2). The mean length of the transcripts was 610 bp. The length distribution is shown in the Figure 2A. More than 96% of the unigenes had a length between 201 and 2500. The N50 for the assembly was determined to be 924 bp, meaning that 50% of the summed size of the assembly was contained in contigs that were at least 924 bp.

**Table 2:** Assembly statistics of the *T. pityocampa* transcriptome.

Nr of unigenes	152,669
Nr of transcripts	161,682
Average (median) transcript length	610 (322)
Min-Max transcript length	201–49,848
N50 transcript length ^a^	924
Total nr of residues	98,648,698

^a^ N50: contig length for which half of the summed size of the assembly is this size or longer.

When compared to previous transcriptome [24], the number of assembled unigenes was increased by more than ten times mainly due to the use of different sequencing platform (Table S2). Illumina sequencing offers significantly higher throughput (greater nucleotide coverage and higher number of reads) than 454-FLX, however it is often criticized for short reads length negatively affecting to the average length of the assembled contigs. In our study, the N50 transcript length was relatively long (924N) although smaller than previous (1517) in 454-FLX and Sanger assembly [24], probably due to the very high sequence coverage and to the addition of the previously published reads.

More than 97% of the 12,000 unigenes obtained previously are included in the new reference transcriptome. The remaining 3% may result from the different assembly methods used in both studies as well as population polymorphisms. Illumina assemblies were previously shown to perform best for *de novo* transcriptome characterization in terms of contig length, transcriptome coverage, and complete assembly of gene transcripts [52].

**Figure 3:**
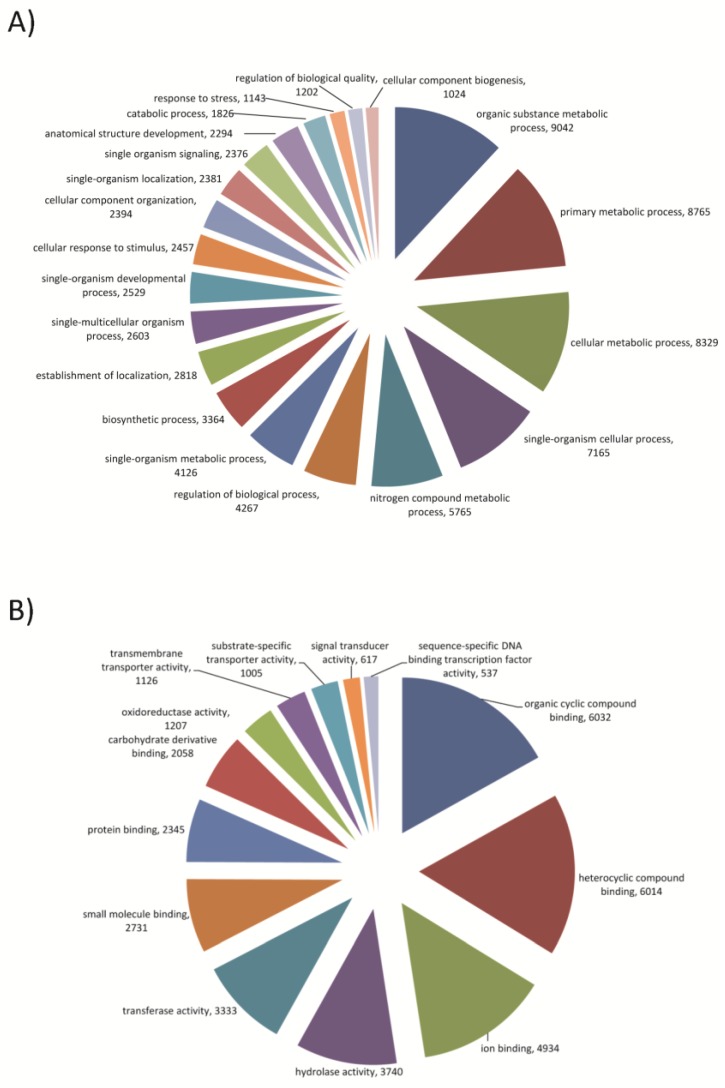
Gene ontology (GO) assignments for *T. pityocampa* transcriptome. Unigenes were classified into functional groups based on level 3 GO assignments as predicted for their involvement in (**A**) biological process and (**B**) molecular function. The number of unigenes assigned to each GO term is shown. We included classes that represent more than 1% of the total number of classified sequences, to simplify the visualization of the results.

For the new reference transcriptome of *T. pityocampa*, 15% (24,442) of the transcripts had homologues in NR protein database (Table S3). We used restrictive parameters to avoid the occurrence of false homologs. A majority of the best hits found in NR belonged, as expected, to insect sequences. Blast2GO was used to assign gene ontology (GO) terms to the predicted proteins derived from the new transcriptome. 18,190 transcripts were assigned to one or more GO category. The number of GO terms per transcript ranged from 1 to 123. Over 90% of the transcripts were assigned between 1–10 GO terms (Figure S1). In total 97,803 GO terms were retrieved and classified according to three categories, biological process (P), molecular function (F) and cellular component (C). The distribution of annotated unigenes across different GO levels in the three main GO domains (Figure 2B) showed that they are concentrated in levels 6–10 for P, 2–9 for F and 2–11 for C, indicating a good accuracy of annotation. The deeper the level, the more precise is the term, but there are fewer genes with annotations at deeper levels [53]. GO level 3 is a suitable compromise between information quality and the number of annotated genes, therefore we determined the classification of GO terms into biological process and molecular function at level 3 (Figure 3).

The most abundant GO terms in P categories were those representing metabolic processes (organic substance metabolic processes, primary metabolic processes and cellular metabolic processes) (Figure 3A). For the F categories the most abundant GO were organic cyclic and heterocyclic compound binding, ion binding and hydrolase activity (Figure 3B). In summary, the broad diversity in GO annotation reflects a broad diversity of sequenced transcriptome. All GO terms are accessible in the Table S4.

**Figure 4:**
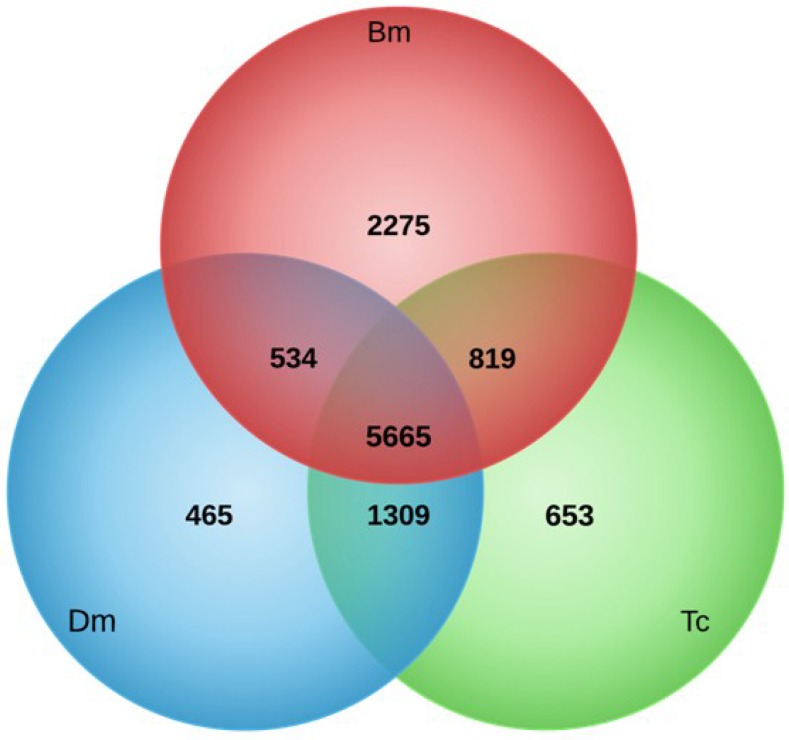
Venn diagram showing the number of orthologs shared between *T. pityocampa* and *B. mori* (Bm), *D. melanogaster* (Dm), and *T. castaneum* (Tc).

We additionally characterized the newly generated transcriptome by analyses of unigenes with orthologs in other insect species: *B. mori*, *D. melanogaster* and *T. castaneum*. Bidirectional blast comparisons against SilkDB, Flybase, and Beetlebase identified 11,720 unigenes that have orthologs in the abovementioned insect species. From them, 9293 unigenes have orthologs in *B. mori*, 7973 unigenes have orthologs in *D. melanogaster* and 8446 unigenes have orthologs in *T. castaneum* (Figure 4). The lists of unigenes with orthologs in the three species are given in the supplementary data Table S5. As expected, the highest number of orthologs were shared with the other Lepidopteran species, *B. mori* (9293). However, similar numbers of orthologs shared with species representing Diptera and Coleoptera (7973 and 8446, respectively) suggests that these set of unigenes might represent a set of homologous genes conserved among insects.

### 3.2. Identification of Viral Sequences

Transcriptome mining for viral-related sequences detected a number of sequences among the *T. pityocampa* unigenes potentially belonging to viral pathogens. Many of the unigene sequences showed similarity to viral sequences due to the presence of functional motifs present in both viral and non-viral sequences or to sequence similarity with retrotransposon elements. The unigenes were manually filtered by length and e-value, and only those sequences with high e-values and several hits from the same type of virus were further analysed. The remaining unigenes with similarity to viral sequences are shown in Table 3 allowing the identification of sequences belonging to three different RNA viruses. Presence of these viral sequences was additionally searched in previous transcriptome derived from laboratory populations of *T. pityocampa* from Portugal. Only rhabdovirus-like sequences were identified in this transcriptome. Neither cypovirus nor iflavirus were present in the laboratory populations of pine processionary moth from Portugal.

**Table 3:** Unigenes in the transcriptome showing similarity to sequences from RNA viruses.

Virus	Unigene	Sequence Length	BLAST Match	e-Value
Cytoplasmic polyhedrosis virus	TPUC35905_TC01	4102	*Heliothis armigera* CPV5 major core protein	0.0
	TPUC31106_TC01	3898	*Orgyia pseudotsugata* CPV5 minor capsid protein	0.0
	TPUC32941_TC01	3698	*Orgyia pseudotsugata* CPV5 RdRp	0.0
	TPUC35768_TC01	3304	*Orgyia pseudotsugata* CPV5 Structural protein	0.0
	TPUC32710_TC01	2780	*Heliothis armigera* CPV5 Rna 5 protein	0.0
	TPUC25109_TC01	1813	*Orgyia pseudotsugata* CPV5Rna 6 protein	0.0
	TPUC37981_TC01	1914	*Orgyia pseudotsugata* CPV5 Viral structural protein 4 (SP4)	0.0
	TPUC80863_TC01	1249	*Heliothis armigera* CPV5 Structural protein (SP)	0.0
	TPUC98042_TC01	1058	*Heliothis armigera* CPV5 Non-structural protein (NSP)	0.0
	TPUC71859_TC01	924	*Orgyia pseudotsugata* CPV5 Polyhedrin (PH)	1.60E-175
Iflavirus	TPUC51699_TC01	9816	*Antheraea pernyi* iflavirus polyprotein	0.0
	TPUC109136_TC01	357	*S.exigua* iflavirus 2 polyprotein	3.30E-58
	TPUC143735_TC01	335	*Antheraea pernyi* iflavirus polyprotein	2.35E-18
	TPUC71578_TC01	328	*S.exigua* iflavirus 2 polyprotein	1.26E-49
	TPUC147958_TC01	323	*Antheraea pernyi* iflavirus polyprotein	1.15E-07
	TPUC03472_TC01	298	*S.exigua* iflavirus 2 polyprotein	9.18E-56
	TPUC100622_TC01	230	*S.exigua* iflavirus 2 polyprotein	3.18E-39
	TPUC83511_TC01	225	*S.exigua* iflavirus 2 polyprotein	1.82E-37
	TPUC151007_TC01	217	*S.exigua* iflavirus 2 polyprotein	3.84E-23
	TPUC91335_TC01	214	*S.exigua* iflavirus 2 polyprotein	1.36E-37
Rhabdovirus	TPUC44929_TC01	4501	Maraba virus L polymerase protein	0.0
	TPUC38841_TC01	2830	*Spodoptera frugiperda* rhabdovirus P protein	6.50E-11
	TPUC14459_TC01	1900	Jurona virus L polymerase protein	1.09E-115
	TPUC75494_TC01	1016	Dolphin rhabdovirus L polymerase protein	7.40E-12
	TPUC37175_TC01	914	*Muscina stabulans* sigmavirus RdRp	5.31E-48
	TPUC48042_TC01	895	*Drosophila immigrans* sigmavirus RdRp	1.35E-15
	TPUC98122_TC01	631	*Muscina stabulans* sigmavirus RdRp	1.51E-55
	TPUC56532_TC01	516	China fish rhabdovirus L polymerase protein	7.95E-72
	TPUC99390_TC01	361	*Muscina stabulans* sigmavirus RdRp	2.79E-34

#### 3.2.1. *T. pityocampa* Cytoplasmic Polyhedrosis Virus, TpCPV 5

**Figure 5:**
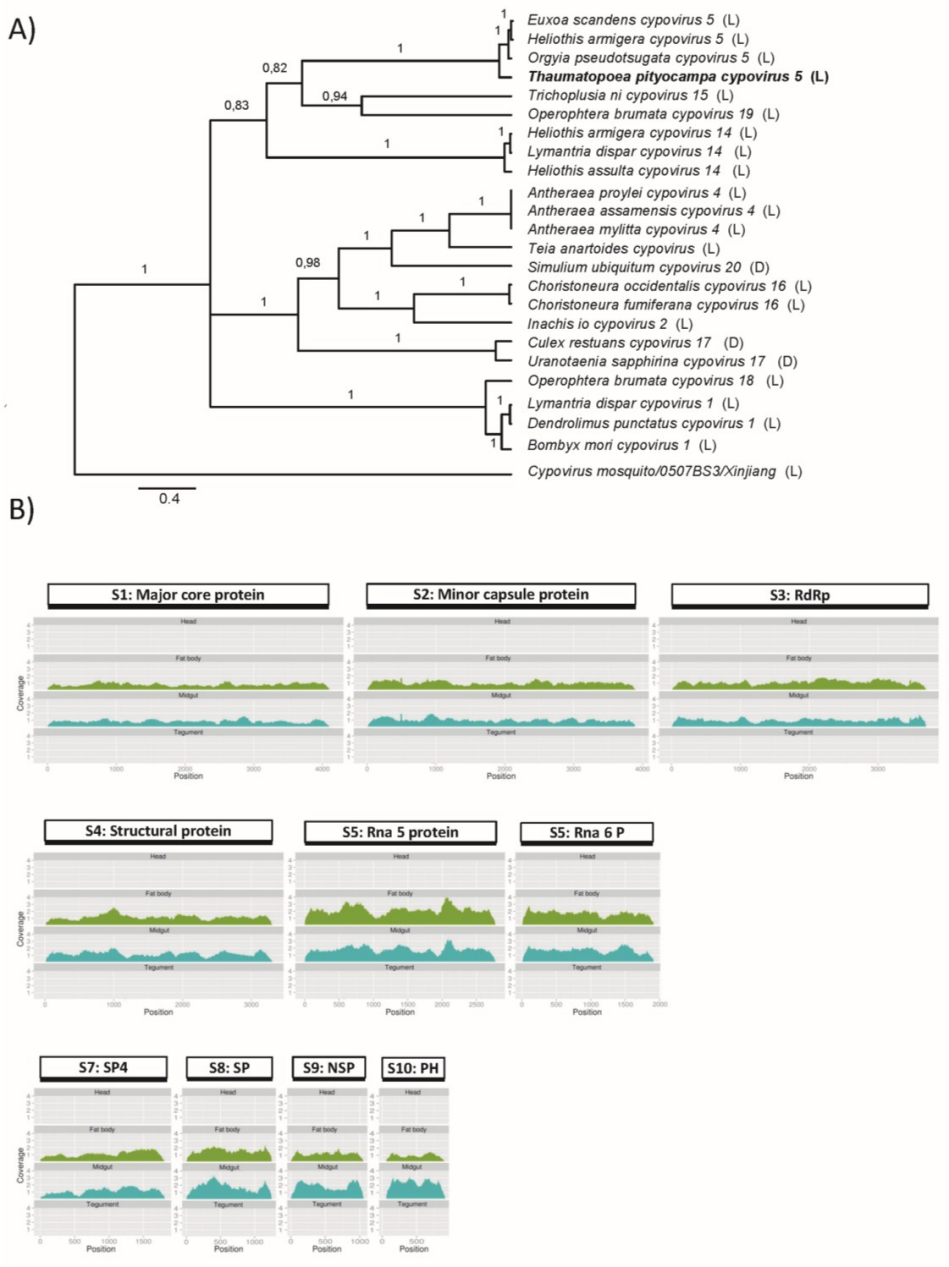
*T. pityocampa* cypovirus 5 phylogenetic relationship, genome structure, and tissue distribution mapping. (**A**) Inferred Bayesian phylogenetic tree based on nucleotide sequence of the polyhedrin gene (segment 10, 1071 nt) within Cypovirus species described. Posterior probabilities are indicated in branches. Scale bar indicates distance in nucleotide substitution/position. Host species order is shown between brackets (D: Diptera, L: Lepidoptera). (**B**) Schematic representation of *T. pityocampa* cypovirus 5 genomic structure including virus reads mapping in head, fat body, midgut and tegument (below). Segments are drawn to scale. Coverage histograms were obtained from BAM files after normalizing by the millions of reads obtained in each library.

We identified 10 unigenes in the *T. pityocampa* transcriptome with significant sequence similarity to genes from cytoplasmic polyhedrosis viruses (cypoviruses, CPVs). The segments are complete and no mutations introducing premature stop codon have been identified. CPVs belong to the genus Cypovirus in the family *Reoviridae* [54]. In the majority of cases cypovirus genomes are composed of 10 double stranded RNA (dsRNA) segments, however a few CPVs contain 11 segments, *i.e.*, *Trichoplusia ni* cypovirus 15 [33]. CPVs form occlusion bodies (polyhedra) and their virions are embedded in polyhedrin, a protein matrix that forms a major part of the occlusion body. CPVs polyhedra may exhibit an icosahedral, cubic, or irregularly-shaped morphology. They dissolve in the high pH of the host midgut, similarly to baculoviruses, and infect primarily midgut cells. CPVs have been isolated from more than 250 insect species [32], mainly Lepidoptera, but also from Diptera, Hymenoptera, Coleoptera and Neuroptera [55,56]. Currently 16 cypovirus species are recognized by the International Committee for Taxonomy of Viruses [33]. Previously, a CPV was described by the group in the Karadeniz Technical University, from larvae of PPM collected in the Samsun, the Black Sea region of Turkey, in 2004–2005 [11]. It showed some similarity in its electropherotype to CPV 5, however a conclusive identification of species could not have been performed until the availability of sequence data. Phylogenetic comparison of the CPV identified in the presented transcriptome revealed also its close relation to species of Cypovirus type 5, grouping in the same clade than other cypoviruses infecting lepidopteran species: *Heliothis armigera* CPV 5 and 8, *Euxoa scandens* CPV 5 or *Orgyia pseudotsugata* CPV 5 (Figure 5A). Accordingly we decided to name this virus as TpCPV 5 (NCBI acc. numbers: KP217033-KP217042).

The genome of TpCPV 5 identified in the transcriptome presented here is composed of 10 segments of 4102, 3898, 3698, 3304, 2780, 1813, 1249, 1058 and 924 bp, from segment 1 to 10, respectively (Figure 5B). These segment sizes are in accordance to the segment sizes estimated by agarose gel electrophoresis of the CPV from *T. pityocampa* described by Ince *et al.* [11], suggesting that the obtained sequences represent the complete genome sequence of another isolate of the TpCPV 5.

Transcriptional analysis of the viral abundance in the four studied tissues showed that TpCPV 5 was present in similar levels in the gut and fat body samples, and completely absent in the head and tegument (Figure 5B). These results suggest that TpCPV 5 is present and probably replicates in both types of tissues. In general, cypovirus infection in the larvae is restricted to the columnar epithelial and to less extent goblet cells in the midgut and fat body [33,57]. Comparison of the coverage profile in midgut and fat body reveals similar abundance for all the 10 fragments. Assuming that the segments of the genome are transcribed at different frequency in Reoviruses (Mertens *et al.*, 2004), these results suggest that our sequences are derived from viral forms and not from active transcription. We cannot discard that viral particles produced in the larval gut were trans-located to the fat body tissue but given the similar abundance in both tissues it is more likely that TpCPV 5 was produced in both tissues. Although additional studies will be needed to confirm that, it seems that TpCPV 5, in contrast to most of CPVs, can actively replicate in fat body.

#### 3.2.2. *T. pityocampa* Iflavirus, TpIV1

**Figure 6:**
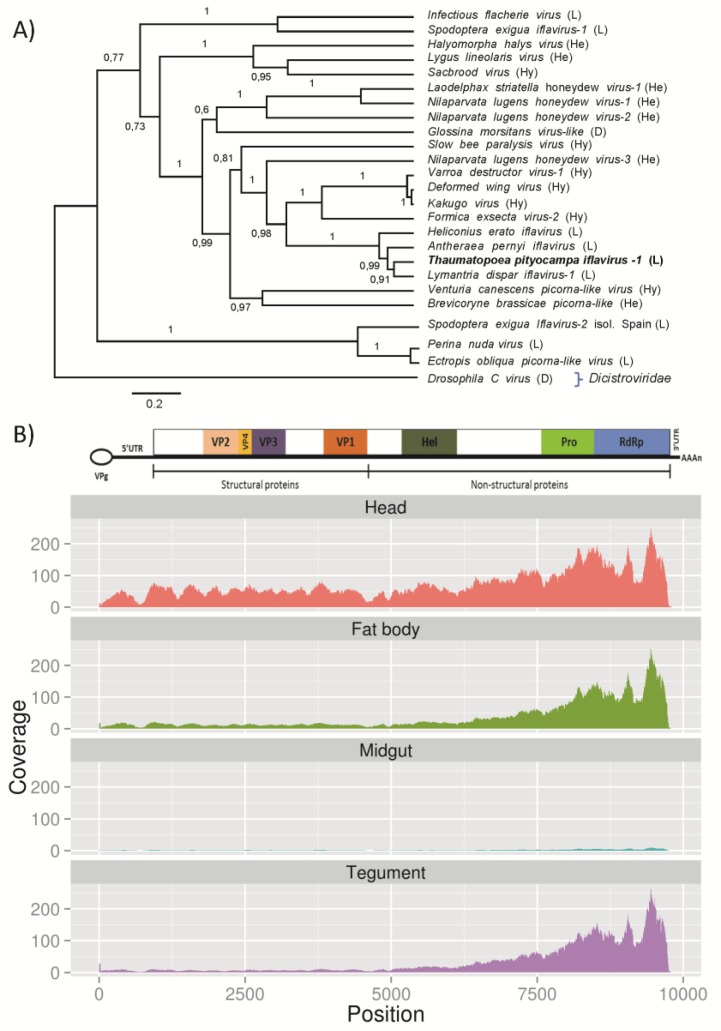
*Thaumatopoea pityocampa* iflavirus −1 phylogenetic relationship, genome structure, and tissue distribution mapping. (**A**) Inferred Bayesian phylogenetic tree based on the amino acid sequences of the RdRp comprising the conserved domains I to VIII (359 aa) of members of the family *Iflaviridae*. *Drosophila C virus* from the family *Dicistroviridae* has been used as outgroup. Posterior probabilities are indicated in branches. Scale bar indicates distance measured as the number of amino acid substitutions per position. Host species order is shown between brackets (Hy: Hymenoptera, He: Heteroptera, D: Diptera, L: Lepidoptera, O: Orthoptera). (**B**) Schematic genome representation of *Thaumatopoea pityocampa* iflavirus −1 including virus reads mapping in head, fat body, midgut and tegument (below). The conserved domains for the helicase (Hel), protease (Pro) and RNA-dependent RNA polymerase (RdRp) are indicated. Limits of the VP1–VP4 polypeptides were predicted by comparison with other iflaviruses. Hypothetical binding of the small viral protein VPg has been included in the scheme. Coverage histograms were obtained from BAM files after normalizing by the millions of reads obtained in each library.

Ten *T. pityocampa* unigenes showing similarity to iflavirus sequences were identified (Table 3). The iflavirus sequence has a similar length that of other iflavirus genomes and no frameshift mutations have been identified. The family *Iflaviridae* is a new member within the order *Picornavirales,* classified by ICTV [58] and currently includes the sole genus *Iflavirus* comprising nine species infecting insects from the orders Lepidoptera, Hymenoptera, Hemiptera and Diptera. Virions are not enveloped and exhibit an icosahedral symmetry with a diameter of about 30 nm [59]. Sequence analysis and comparisons of the obtained unigenes to known iflaviruses revealed that entire genome sequence of a new iflavirus was present in the studied transcriptome (TPUC51699_TC01). As this is the first iflavirus described in this insect species we name it *T. pityocampa* iflavirus 1 (TpIV1) (NCBI acc. number: KP217032). Phylogenetic analysis based on the RdRp domain showed that TpIV1 is most closely related to *Lymantria dispar* iflavirus 1 (LdIV1) (Figure 6A) isolated from the forest pest, *Lymantria dispar* (Gypsy moth). Members of the *Iflaviridae* family fall into two lineages; one grouping viruses with similarity to *Iflaviridae* type species, *Infectious flacherie virus* (IFV) and second, composed of only three viruses so far, *Perina nuda* virus (PvV), *Ectropis obliqua* picorna-like virus (EoPV) and *Spodoptera exigua* iflavirus-2 (SeIV-2). TpIV1, like most of known iflaviruses, groups together with the IFV. The genome structure of TpIV1 is in accordance with other iflavirus genomes. It is 9816 nt long and encodes four structural proteins (VP1-VP4) headed by the leader peptide (L) on the 5′end and three non-structural proteins, helicase, protease and RNA dependent RNA polymerase, on the 3′end. The coding region is flanked by untranslated regions (UTR) at both ends. The 5′UTR was predicted to be 597 nt long, and 3′UTR was predicted to be 183 nt long, together accounting for 8% of the genome (Fig 6B). The iflavirus 5′UTR lengths range between 390 nt (PnV) and 1156 nt (Deformed wing virus, DWV). Long 5′UTRs in many iflaviruses possess a functional role as internal ribosome entry site (IRES), for translation initiation [60,61]. It remains to be studied if TpIV1 5′UTR forms functional IRES structures. The monocistronic genome contains one large uninterrupted ORF that translates into 2958 aa polyprotein. The TpIV1 polyprotein shows 70% identity to *Lymantria dispar* iflavirus 1 (LdIV1) polyprotein. Sequence information did not reveal the presence of mutations that introduce premature stop codons or changes in the reading frame supporting that TpIV-1 is able to replicate and establish infection in *T. pityocampa*. Nevertheless, further experiments are needed to confirm that TpIV1 produces infectious particles.

Transcriptional analysis of the TpIV1 abundance in the four studied tissues showed, to our surprise, that TpIV1 was most abundant in larval heads (Figure 6B). TpIV1 transcripts were also present in fat body and integument, and in lower abundance in gut tissue. Studies on other iflaviruses show that they present a wide range of tissue tropism. DWV infection can spread to whole body of the bee including queen ovaries, fat body and drone seminal vesicles [62]. *Spodoptera exigua* iflaviruses (SeIV1 and SeIV2) were detected in all three tested tissues, midgut, hemocytes and fat bodies; however SeIV1 showed tropism to larval midgut [63]. LdIV1 was shown to be present in all four tested tissues, ovarioles, hemocytes, midgut and fat body [64]. *Heliconius erato* iflavirus (HeIV) genome was obtained from the transcriptome of *Heliconius* butterflies assembled from libraries of four tissues, antennas, mouth parts, heads and legs, suggesting that this virus is present in at least one of these tissues [65]*.* *Antheraea pernyi* iflavirus (ApIV), causing a vomiting disease in Chinese oak silkmoth was detected in all developmental stages, but its tissue distribution has not yet been determined [60]. Presence of iflavirus in heads was reported for Kakugo virus (KV)/DWV in bees [66,67]. Pathological effect produced by iflaviruses can vary depending on the host-virus combination. For instance, IFV that is mainly present in the midgut cells from the silkworm larvae produces lethal diarrhea [68,69], in contrast KV is mainly found in the brain of aggressive honey bees [70], although correlation between KV presence and aggressiveness has not been established.

#### 3.2.3. *T. pityocampa* Rhabdovirus-Like Sequences

**Figure 7:**
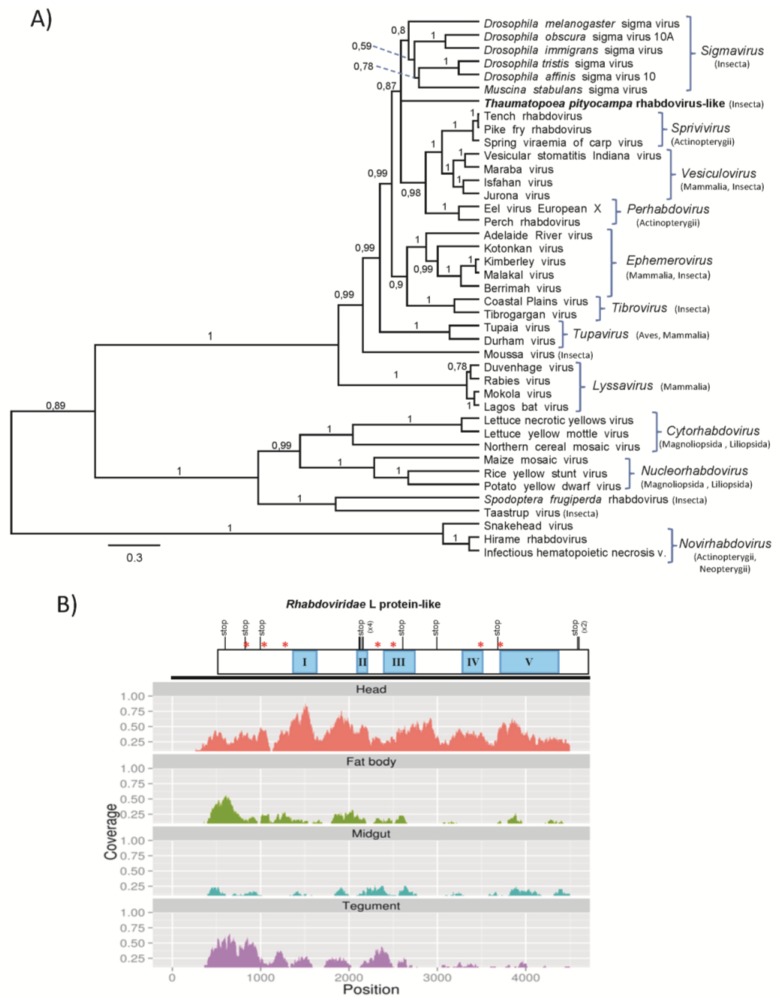
Phylogenetic relationships, genome structure, and tissue distribution mapping of rhabdovirus-like sequences detected in *Thaumatopoea pityocampa*. (**A**) Molecular phylogenetic analysis among the family *Rhabdoviridae* of the predicted *Thaumatopoea pityocampa* rhabdo-like virus, based on alignment of the 158-residue of domain III L-protein by Bayesian method based on WAG model. Posterior probabilities are indicated in branches. Scale bar indicates an evolutionary distance measured as the number of amino acid substitutions per position. Host species class is shown between brackets. (**B**) Schematic representation of the contig enclosing the L-protein sequence from the *Thaumatopoea pityocampa* rhabdo-like virus including reads mapping in four studied tissues, head, fat body, midgut and tegument (below). Asterisks are indicative of Indel mutations introducing frameshift; stop indicates premature termination codons. Coverage histograms were obtained from BAM files after normalizing by the millions of reads obtained in each library.

A few *T. pityocampa* unigenes showed similarity to rhabdovirus sequences (Table 3). The same rhabdovirus-like sequences have been identified in the 454 transcriptome from Portuguese populations of *T. pityocampa*. Rhabdoviruses genomes typically consist of five structural protein genes organized in the following order: 3′- N-P-M-G-L-5′. N, P and L proteins together with genomic RNA form a ribonucleoprotein complex for replication and transcription, while M and G proteins are structural proteins of the virion [71]. Blastx analysis showed that most of them showed similarity to rhabdovirus L protein, and one unigene showed similarity to P protein from Sf-rhabdovirus with 38% identity in a fragment of 410 nt. The phylogenetic analysis based on a conserved 158-amino acids fragment included in the domain III [39] of the L protein, revealed that Tp rhabdovirus-like unigene might be related to rhabdoviruses infecting fish or *Drosophila* sigmaviruses, however it does not group with any phylogenetic clade (Figure 7A). Low bootstrap values indicate that Tp rhabdovirus-like unigene is distantly related to other rhabdoviruses, and its phylogenetic status may change once the entire genome sequence will be available. L proteins of rhabdoviruses share six conserved domains [72] and domain V identified also in Tp rhabdovirus-like sequence (Figure 7B) is believed to play an essential role of RdRp activity, *i.e.*, capping mRNA. P protein (phosphoprotein) forms a small subunit of RdRp and serves as a transcription factor.

Tp rhabdovirus-like sequence identified in the presented transcriptome, lack domain VI from the L protein and also contains an important number of mutations that introduce premature termination of translation which are indicative of the lack of selective pressure. Moreover, mapping the rhabdovirus-like sequence shows low coverage when compared to cypovirus and iflavirus (Figure 7B). This suggests that identified sequences may represent rhabdovirus-like non-retroviral integrated RNA viruses (NIRV) in the genome of *T. pityocampa*. NIRVs although transcribed, do not represent an actively replicating virus. Recently, rhabdovirus-like NIRVs have been identified within several *Drosophila* species [73], and also in ticks [74] and mosquitoes [75]. Most NIRVs are pseudogenes; however some have complete open reading frames (ORFs) and are expressed as RNA [76,77].

The presence of rhabdovirus-like pseudogenes in *T. pityocampa* transcriptome is indicative of the possible existence of rhabdoviruses infecting this species. Sequence information reported here may assist for the identification of infectious rhabdoviruses that could be deployed against *T. pityocampa*. Rhabdoviruses are ubiquitous in nature and possess a very wide host range, including vertebrates, invertebrates and plants [78]. A few vertebrate rhabdoviruses were described from insects as vectors for transmission, while very few rhabdoviruses were directly isolated from insects as their unique hosts [79]. Rhabdoviruses (sigma viruses) have been also described from several *Drosophila* spp. [80,81,82]. These vertically transmitted *Drosophila* sigma viruses form a deep-branching clade within the *Rhabdoviridae* suggested to be recognized as a new genus [81]. Recently, the first rhabdovirus infecting a lepidopteran host was identified. Sf-rhabdovirus was found permanently infecting insect cell line from *Spodoptera frugiperda*, Sf9 [83]. Sf-rhabdovirus was more closely related to plant rhabdoviruses than to invertebrate rhabdoviruses and phylogenetically grouped with the Taastrup virus, a recently isolated rhabdovirus from leafhopper (Hemiptera) [84].

## 4. Conclusions

Pine processionary moth causes significant damage to pinewoods, but its occurrence is also an issue for public and animal health. Using NGS on different tissues from larvae collected in 12 distinct geographical locations we have generated a reference transcriptome of the PPM for further physiological as well as ecological studies that could help to develop novel methods for the control of this pest. Our study has also allowed the identification and genomic sequencing of potential viral pathogens naturally infecting PPM larvae. Further studies to confirm the identity, infectivity and host range of the putative viruses would contribute to confirm their potential as pest control agent. Nevertheless, this study will contribute to the understanding of the dynamics of PPM in the field and to the development of viral agents for a cost-effective reduction of the damage produced by this pest.

## Supplementary Files

Supplementary File 1
