# Supplementary material for: In Search of Pathogens: Transcriptome-Based Identification of Viral Sequences from the Pine Processionary Moth (Thaumetopoea pityocampa)
Source: Viruses. 2015 Jan 23;7(2):456–79. doi: 10.3390/v7020456 (PMC4353898; doi:10.3390/v7020456)
Supplement: Supplementary File 1 [file viruses-07-00456-s001.pdf]

Supplementary Files

Table S1. Accession numbers of viral sequences used in these work for sequence analyses and phylogenetic reconstruction.

Name	GeneBank Accession	Family
Drosophila C virus	AF014388.1	*Dicistroviridae*
Infectious flacherie virus	AB000906.1	*Iflaviridae*
Spodoptera exigua iflavirus-1	JN091707.1	*Iflaviridae*
Spodoptera exigua iflavirus-2 isolate Spain	KJ186788	*Iflaviridae*
Lygus lineolaris virus-1	JF720348.1	*Iflaviridae*
Sacbrood virus	AF092924.1	*Iflaviridae*
Glossina morsitans virus-like	EZ407281.1	*Iflaviridae*
Slow bee paralysis virus	EU035616.1	*Iflaviridae*
Brevicoryne brassicae picorna-like virus	EF517277.1	*Iflaviridae*
Varroa destructor virus-1	AY251269.2	*Iflaviridae*
Deformed wing virus	AJ489744.2	*Iflaviridae*
Kakugo virus	AB070959.1	*Iflaviridae*
Perina nuda virus	AF323747.1	*Iflaviridae*
Ectropis obliqua picorna-like virus	AY365064.1	*Iflaviridae*
Nasonia vitripennis virus-1	FJ790486.1	*Iflaviridae*
Halyomorpha halys virus	AGY34702.1	*Iflaviridae*
Laodelphax striatella honeydew *virus-1*	AHK05791.1	*Iflaviridae*
Nilaparvata lugens honeydew virus-1	BAN19725.	*Iflaviridae*
Nilaparvata lugens honeydew virus-2	BAN57352.1	*Iflaviridae*
Nilaparvata lugens honeydew virus-3	BAN57353.1	*Iflaviridae*
Formica exsecta virus-2	AHB62422.1	*Iflaviridae*
Heliconius erato iflavirus	AHW98099.1	*Iflaviridae*
Antheraea pernyi iflavirus	YP_009002581.1	*Iflaviridae*
Lymantria dispar iflavirus-1	AIF75200.1	*Iflaviridae*
Venturia canescens picorna-like virus	AY534885.1	*Iflaviridae*
Antheraea mylitta cypovirus	AY212272.1	*Reoviridae*
Antheraea assamensis cypovirus	AY212275.1	*Reoviridae*
Antheraea proylei cypovirus	AY212276.1	*Reoviridae*
Bombyx mori cypovirus 1	D37768.1	*Reoviridae*
Choristoneura fumiferana cypovirus	U95954.1	*Reoviridae*
Choristoneura occidentalis cypovirus 16	EU201043.1	*Reoviridae*
Culex restuans cypovirus	DQ212785.	*Reoviridae*
Lymantria dispar cypovirus 1	NC_003025.1	*Reoviridae*
Cypovirus mosquito/0507BS3/Xinjiang	FJ150869.1	*Reoviridae*
Dendrolimus punctatus cypovirus 1	AY211094.1	*Reoviridae*
Euxoa scandens cypovirus	J04338.1	*Reoviridae*
Heliothis armigera cypovirus 14	DQ010323.1	*Reoviridae*
Heliothis armigera cypovirus 5	DQ077912.1	*Reoviridae*
Heliothis assulta cypovirus 14	DQ077914.1	*Reoviridae*
Inachis io cypovirus 2	KC588375.1	*Reoviridae*
Lymantria dispar cypovirus 1	AF389471.1	*Reoviridae*
Lymantria dispar cypovirus 14	AF389461.1	*Reoviridae*

Table S1. *Cont.*

Name	GeneBank Accession	Family
Operophtera brumata cypovirus 18	DQ192250.1	*Reoviridae*
Operophtera brumata cypovirus 19	DQ192254.1	*Reoviridae*
Orgyia pseudotsugata cypovirus 5	KC588365.1	*Reoviridae*
Simulium ubiquitum cypovirus	DQ834386.1	*Reoviridae*
Teia anartoides cypovirus	HQ901376.1	*Reoviridae*
Trichoplusia ni cypovirus 15	AF291692.1	*Reoviridae*
Uranotaenia sapphirina cypovirus	AY876384.1	*Reoviridae*
Adelaide River virus	AAG10421.1	*Rhabdoviridae*
Lettuce necrotic yellows virus	YP_425092.1	*Rhabdoviridae*
Lettuce yellow mottle virus	ABV56129.1	*Rhabdoviridae*
Northern cereal mosaic virus	NP_597914.1	*Rhabdoviridae*
Kotonkan virus	AEI17640.1	*Rhabdoviridae*
Kimberley virus	AFR67096.1	*Rhabdoviridae*
Malakal virus	AFR67117.1	*Rhabdoviridae*
Berrimah virus	AEH58025.1	*Rhabdoviridae*
Rabies virus	NP_056797.1	*Rhabdoviridae*
Lagos bat virus	ABZ81161.1	*Rhabdoviridae*
Duvenhage virus	ABZ81216.1	*Rhabdoviridae*
Mokola virus	AGQ16857.1	*Rhabdoviridae*
Infectious hematopoietic necrosis virus	NP_042681.1	*Rhabdoviridae*
Hirame rhabdovirus	NP_919035.1	*Rhabdoviridae*
Snakehead virus	NP_050585.1	*Rhabdoviridae*
Rice yellow stunt virus	NP_620502.1	*Rhabdoviridae*
Maize mosaic virus	YP_052855.1	*Rhabdoviridae*
Potato yellow dwarf virus	ADE45274.1	*Rhabdoviridae*
Eel virus European X	CBH20130.1	*Rhabdoviridae*
Perch rhabdovirus	AFX72892.1	*Rhabdoviridae*
Muscina stabulans sigmavirus	AEA49879.1	*Rhabdoviridae*
Drosophila immigrans sigmavirus	AEA49878.1	*Rhabdoviridae*
Drosophila obscura sigmavirus 10A	ACU65444.1	*Rhabdoviridae*
Drosophila melanogaster sigmavirus AP30	CBA18272.1	*Rhabdoviridae*
Drosophila tristis sigmavirus	AEA49876.1	*Rhabdoviridae*
Drosophila affinis sigmavirus 10	ACU65445.1	*Rhabdoviridae*
Pike fry rhabdovirus	ACP28002.1	*Rhabdoviridae*
Spring viraemia of carp virus	ABF06664.1	*Rhabdoviridae*
Tench rhabdovirus S64	AGE10373.1	*Rhabdoviridae*
Coastal Plains virus	ADG86364.1	*Rhabdoviridae*
Tibrogargan virus	ADG86355.1	*Rhabdoviridae*
Durham virus	ADB88761.1	*Rhabdoviridae*
Tupaia virus	AAX47602.1	*Rhabdoviridae*
Maraba virus	AEI52253.1	*Rhabdoviridae*
Isfahan virus	YP_007641386.1	*Rhabdoviridae*
Vesicular stomatitis Indiana virus	AAA48371.1	*Rhabdoviridae*
Jurona virus	AEG25349.1	*Rhabdoviridae*
Spodoptera frugiperda rhabdovirus	AHN92647.1	*Rhabdoviridae*
Taastrup virus	AY423355.1	*Rhabdoviridae*
Moussa virus	ACZ81402.1	*Rhabdoviridae*

Table S2. Comparison of previous (Gschloessl *et al.*, 2014) and present transcriptome sequencing and assembly.

Nr of transcripts in 454 transcriptome [a]	12,011
Nr of transcripts in present transcriptome [b]	161,682
Nr of transcripts in present transcriptome that blast with 454 transcriptome	17,273
Nr of new transcripts in present transcriptome when compared to 454 transcriptome	144,409
Nr of transcripts from 454 transcriptome in present transcriptome	11,680

[a] Gschloessl *et al*., 2014. [b] Illumina and 454 transcriptome assembly.
